# Tryptophan nitration of immunoglobulin light chain as a new possible biomarker for atopic dermatitis

**DOI:** 10.3164/jcbn.18-53

**Published:** 2018-09-15

**Authors:** Kyoichi Iizumi, Hiroaki Kawasaki, Ayako Shigenaga, Mitsutoshi Tominaga, Ayaka Otsu, Atsuko Kamo, Yayoi Kamata, Kenji Takamori, Fumiyuki Yamakura

**Affiliations:** 1 Juntendo University Faculty of Health and Sports Science, 1-1 Hirakagakuendai, Inzai, Chiba 270-1695, Japan; 2 Institute for Environmental and Gender-Specific Medicine, Juntendo University Graduate School of Medicine, 2-1-1 Tomioka, Urayasu, Chiba 279-0021, Japan; 3 Institute of Health Sports Science & Medicine, Juntendo University, 1-1 Hirakagakuendai, Inzai, Chiba 270-1695, Japan; 4 Juntendo University Faculty of Healthcare and Nursing, 2-1-1 Takasu, Urayasu, Chiba 279-0023, Japan; 5 Juntendo University Faculty of International Liberal Arts, 2-1-1, Bunkyo, Hongo, Tokyo 113-8424, Japan

**Keywords:** atopic dermatitis, biomarker, 6-nitrotryptophan, NC/Nga mouse, oxidative stress

## Abstract

To reduce the incidence and severity of atopic dermatitis, detection and treatment at an early stage are urgently required, but no effective biomarker has been reported. In this study, we attempted to detect a candidate biomarker of early stage atopic dermatitis by focusing on the levels of nitrated residues in the plasma proteins of atopic dermatitis model mice (NC/Nga mice). We found that the immunoglobulin (Ig) light chain was more highly nitrated in the plasma of the animal model than that of control mice. Western blot analysis showed a statistically significant difference between the 6-nitrotryptophan content of the Ig light chain in the NC/Nga mice before onset of atopic dermatitis symptoms and that of the control mice. LC-ESI-MS/MS analysis demonstrated that these light chains contained nitrotryptophan (Trp56) and nitrotyrosine (Tyr66). Immunofluorescence staining revealed a significant increase in manganese superoxide dismutase and inducible nitric oxide synthase production in the skin lesions of the NC/Nga mice. Furthermore, we found protein-bound 6-nitrotryptophan and 3-nitrotyrosine only in the lesioned skin, where their signals partially overlapped with the IgG signal. Our findings suggest that the 6-nitrotryptophan content of Ig light chains could be a new biomarker for detecting early stage atopic dermatitis.

## Introduction

Atopic dermatitis (AD) is an inflammatory skin disease, the prevalence of which has increased over the last 30 years, such that it now affects 10–20% of the children in developed countries.^(1,2)^ Although the pathology of AD is not fully understood, it is thought to be to associated with epidermal barrier dysfunction and cutaneous immune dysfunction.^(3,4)^ AD causes an intense itch in eczematous lesions, and consequently these lesions are damaged by repeated scratching. The scratching in turn increases the itch further. In this way, an itch-scratch cycle is initiated and develops.^(5)^ Accordingly, treatment of AD at an early stage would be expected to prevent severe cases. Although several biomarkers for AD have been proposed using the serum of AD patients, there are no effective biomarkers to detect AD near or before the onset of skin lesions.^(6)^ Existing biomarkers are not helpful for detecting early stage AD, because they are used to detect disease progression. Therefore, a new early stage biomarker is highly desirable for the prevention of severe AD.

In inflammatory diseases, including AD, the generation of reactive oxygen species (ROS) and reactive nitrogen species (RNS) is increased.^(7,8)^ In these environments, peroxynitrite (ONOO^−^) is formed via the reaction between superoxide anion (O_2_^•−^) and nitric oxide (NO^•^).^(9)^ Peroxynitrite directly causes various modifications of lipids, nucleotides, and proteins. The nitration of the tyrosine residues of proteins, to yield 3-nitrotyrosine (3-NO_2_Tyr), is one such modification, and it has been used as a biomarker for oxidative stress.^(10)^ The nitration of enzymes can result in decreased, or sometimes increased, enzymatic activity.^(11,12)^ In addition, we previously reported that 6-nitrotryptophan (6-NO_2_Trp), the nitration product of tryptophan, is produced in the presence of peroxynitrite.^(13)^

Kawasaki *et al.*^(14)^ revealed that 6-NO_2_Trp and 3-NO_2_Tyr formation is increased in the skin of an animal model for AD (NC/Nga mice) relative to that in the skin of control mice. Interestingly, the 6-NO_2_Trp content of the enzyme carbonic anhydrase III in the model mice was significantly different from that in the control mice even before the NC/Nga mice developed AD-like skin lesions. These results suggested that the amount of 6-NO_2_Trp in proteins could serve as a new biomarker for AD. However, the use of carbonic anhydrase III as a biomarker has several limitations, such as the need for skin biopsy samples from asymptomatic patients before the onset of AD symptoms. Accordingly, minimally invasive biomarkers such as plasma proteins are being sought.

In this study, we attempted to detect a new biomarker for the early stage of AD by focusing on the nitrated residue content of plasma proteins as an indicator. We used NC/Nga mice as an animal model for AD.^(15)^ NC/Nga mice develop AD-like skin lesions when maintained under conventional conditions.^(16–18)^ By using this mouse model, we found that an increase in the 6-NO_2_Trp content of IgG in plasma could serve as a new biomarker for the early stage of AD.

## Materials and Methods

### Animals

We used NC/Nga mice as an animal model for AD. NC/Nga mice develop AD-like skin lesions when kept under conventional conditions for more than 8 weeks, but not when kept under specific-pthogen-free (SPF) conditions. Therefore, we used 10-week-old NC/Nga mice bred under conventional conditions as our animal model for AD (AD-NC/Nga mice). Ten-week-old NC/Nga mice bred under SPF conditions were used as a control (control-NC/Nga mice); these mice showed no AD-like skin lesions. In addition, 5- and 7-week-old NC/Nga mice bred under conventional conditions were used to confirm time-dependent changes in the onset of AD. All mice were purchased from Japan SLC, Inc. (Shizuoka, Japan). The animals were maintained under a 12-h light-dark cycle at 23 ± 1°C. Food and tap water were provided ad libitum. All animal procedures were approved by the Institutional Animal Care and Use Committee at Juntendo University Graduate School of Medicine and Graduate School of Sports and Health Science (H26-07) and conformed to the guidelines for the use of laboratory animals of the National Institutes of Health.

### Plasma preparation

Mice were deeply anesthetized with pentobarbital (somnopentyl; Kyoritu Seiyaku, Tokyo, Japan) or diethyl ether, and then blood was collected from the posterior vena cava by means of a syringe pump containing heparin and cOmplete Protease Inhibitor (Roche Applied Science, Basel, Switzerland). The heparinized blood was centrifuged 800 × *g* for 20 min at 4°C. The plasma was carefully transferred to a new tube and the protein concentrations in the plasma were detected by using the Pierce BCA protein assay kit (Thermo Scientific, Rockford, IL).

### Polyacrylamide gel electrophoresis

Plasma proteins (60 µg) were precipitated by adding three volumes of ice-cold acetone and keeping the samples at −20°C for 1 h. The samples were then centrifuged at 15,000 × *g* for 15 min, and the supernatant was removed. For two-dimensional polyacrylamide gel electrophoresis (2D-PAGE), the pellets was dissolved in rehydration solution containing 8 M urea, 2% CHAPS, 40 mM dithiothreitol (DTT), 0.002% bromophenol blue (BPB), and 0.5% immobilized pH gradient (IPG) buffer (GE Healthcare, Buckinghamshire, UK).^(19)^ This solution containing 60 µg of plasma proteins was loaded onto 7-cm IPG strips (pH gradient 3–10, non-linear, Immobiline DryStrip gels, GE Healthcare) and then subjected to isoelectric focusing (IEF) by using an Ettan IPGphor 3 apparatus (GE Healthcare) under the following conditions: rehydration for 12 h, 300 V for 4 h (step and hold), 1,000 V for 30 min (gradient), 5,000 V for 90 min (gradient), and 5,000 V for 30 min (step and hold) at 20°C. After IEF, the IPG strips were equilibrated for 30 min by using an equilibration solution containing 50 mM Tris (pH 6.8), 6 M urea, 30% glycerol, 2% SDS, 65 mM DTT, and 0.02% BPB. The strips were then embedded in 0.5% agarose on the top of a 10% acrylamide gel. The second-dimension SDS-PAGE was carried out at 20 mA for 1.5 h.

For one-dimensional polyacrylamide gel electrophoresis (1D-PAGE), the plasma samples were mixed with an aliquot of SDS sample buffer (125 mM Tris, pH 6.8, 4% SDS, 10% mercaptoethanol) and heated for 3 min at 95°C, and then applied (2 µg of protein/lane) to a 12.5% acrylamide gel containing a 4.5% stacking gel.^(20)^ Electrophoresis was carried out at 20 mA for 1.5 h.

### Western blot analysis

After 2D-PAGE or 1D-PAGE, the proteins on the gels were electroblotted onto polyvinylidene difluoride membranes (Immobilon-P, 0.45 µm; Millipore-Merk, Darmstadt, Germany) at 500 mA for 2 h by using a Hoefer TE 42 transfer unit (GE Healthcare). Portions of the membranes were stained by SYPRO Ruby (Thermo Fisher Scientific) to detect total proteins. The remaining membranes were blocked with blocking buffer containing 2% gelatin (Sigma-Aldrich, St. Louis, MO) in phosphate-buffered saline containing 0.05% (v/v) Tween 20, pH 7.4 (PBS-T) for 2 h at room temperature. The membranes were then incubated with anti-6-NO_2_Trp monoclonal antibody (1:50,000 dilution) or anti-NO_2_Tyr monoclonal antibody (1:25,000 dilution; TransGenic Inc., Kobe, Japan), which had been conjugated with horseradish peroxidase (HRP) by using the Peroxidase Labeling Kit-SH (Dojindo Molecular Technologies, Inc., Kumamoto, Japan), in 2% gelatin/PBS-T at 4°C overnight. Anti-6-NO_2_Trp monoclonal antibody was prepared as described previously or was a kind gift from the Japan Institute for the Control of Aging, NIKKEN SEIL Co., Ltd, Tokyo, Japan.^(14)^ Signals were detected as chemifluorescence by using Pierce Western Blotting Substrate plus (Thermo scientific, Rockford, IL) and the Typhoon 9400 variable imager (GE Healthcare). To detect immunoglobulin (Ig) light chains, the blotted membranes were washed with Restore PLUS Western Blot Stripping buffer (Thermo Scientific) for 30 min, and then blocked with 2% gelatin/PBS-T. The membranes were incubated with Cy5-conjugated donkey anti-mouse IgG (H&L) (1:1,000 dilution, Jackson ImmunoResearch, West Grove, PA) in 2% gelatin/PBS-T for 2 h at room temperature. The fluorescence signals of Cy5 were detected by using the Typhoon 9400 variable imager. Signal intensities were quantified by using ImageQuant TL ver. 2005 software (GE Healthcare).

### Measurement of plasma IgE concentration

The plasma IgE concentration was measured by using an enzyme-linked immunosorbent assay (ELISA) kit (ab157718, Abcam) in accordance with the manufacturer’s instructions.

### Nanoelectrospray ionization-tandem mass spectrometry

To detect nitrated residues of proteins, the proteins on the 1D-PAGE gels were stained with Coomassie Brilliant Blue R-250 (CBB) (Nakalai Tesque, Kyoto, Japan), and the protein bands corresponding to immunoreactive bands were cut out and digested with trypsin. The tryptic peptides were subjected to LC-ESI-MS/MS analysis using a Thermo Fisher Scientific LXQ mass spectrometer with nano-liquid chromatography (AMR, Inc., Tokyo, Japan). The samples were analyzed as described previously.^(21)^ The conditions for nano-LC were as follows: Zaplous α Pepc18 column (0.1 mm i.d. × 150 mm) and elution with 0.1% formic acid in 2% CH_3_CN (solvent A) and 0.1% formic acid in 90% CH_3_CN (solvent B) using a program of 5% solvent B for equilibration, a gradient of 1.2% solvent B/min for 50 min, and a flow rate of 0.5 µl/min. A database search on Swiss-Prot was performed by using the MASCOT search engine (Matrix Science, UK).

### Immunofluorescence staining

Skin sections were prepared from lesioned skin of 10-week-old NC/Nga mice kept under conventional conditions (AD-NC/Nga mice) and from normal skin of 10-week-old NC/Nga mice kept under SPF conditions (control-NC/Nga mice). The dorsal skin was excised from sacrificed mice and fixed in 4% paraformaldehyde in 0.1 M phosphate buffer (pH 7.4) for 4 h at 4°C.^(22)^ The small pieces of skin were washed with PBS, and then successively immersed in 10%, 15%, and 20% sucrose in PBS. The fixed skin was then embedded in OCT compound (Sakura Finetechnical Co., Ltd., Tokyo, Japan) and frozen. Cryosections (10-µm-thick) were prepared by using a CM1850 cryostat (Leica, Nussloch, Germany) and mounted on silane-coated glass slides. The sections were then immersed in PBS-T containing 5% normal goat serum, 2% bovine serum albumin, and 0.2% Triton X-100 for 1 h at room temperature. They were then incubated with primary antibody for 12 h at room temperature. The primary antibodies used to detect SOD isoforms were as follows: anti-Cu/Zn-SOD antibody (1:50 dilution; Enzo Life Sciences, Farmingdale, NY), anti-Mn-SOD antibody (1:50 dilution; Enzo Life Sciences), and anti-EC-SOD antibody (1:50 dilution; Stressgen Bioreagents, BC, Canada). The primary antibodies used to detect NOS isoforms were as follows: anti-iNOS antibody (1:50 dilution; Abcam, Cambridge, UK), anti-nNOS antibody (1:50 dilution; BD Biosciences, San Jose, CA), and anti-eNOS antibody (1:50 dilution; BD Biosciences). The localization of the nitrated proteins was detected by using an anti-3-NO_2_Tyr antibody (Alpha Diagnostic International, San Antonio, TX) and an anti-6-NO_2_Trp antibody, which was purified from the serum of rabbits immunized with 6-NO_2_Trp-containing peptide as described elsewhere.^(23)^ After three washes, the sections were incubated with Alexa Fluor 488-conjugated-goat anti-rabbit IgG secondary antibodies (1:500 dilution; Jackson ImmunoResearch) for 1 h at room temperature. The sections were then subjected to double immunofluorescence staining using Alexa Fluor 594-conjugated-donkey anti-mouse IgG (H&L) antibody (1:500 dilution; Jackson ImmunoResearch). The sections were mounted in Vectashield mounting medium with 4',6-diamidino-2-phenylindole (DAPI) (Vector Laboratories, Peterborough, UK). Immunoreactivity was confirmed with a confocal laser scanning microscope (LSM 710; ZWISS, Jena, Germany).

### Statistical Analysis

Statistical analysis was performed by using a one-way ANOVA with Dunnett’s post-hoc test in GraphPad PRISM ver. 6.03 (GraphPad Software Inc, San Diego, CA).

## Results

### Detection of nitrated proteins in plasma

**Fig. 1:**
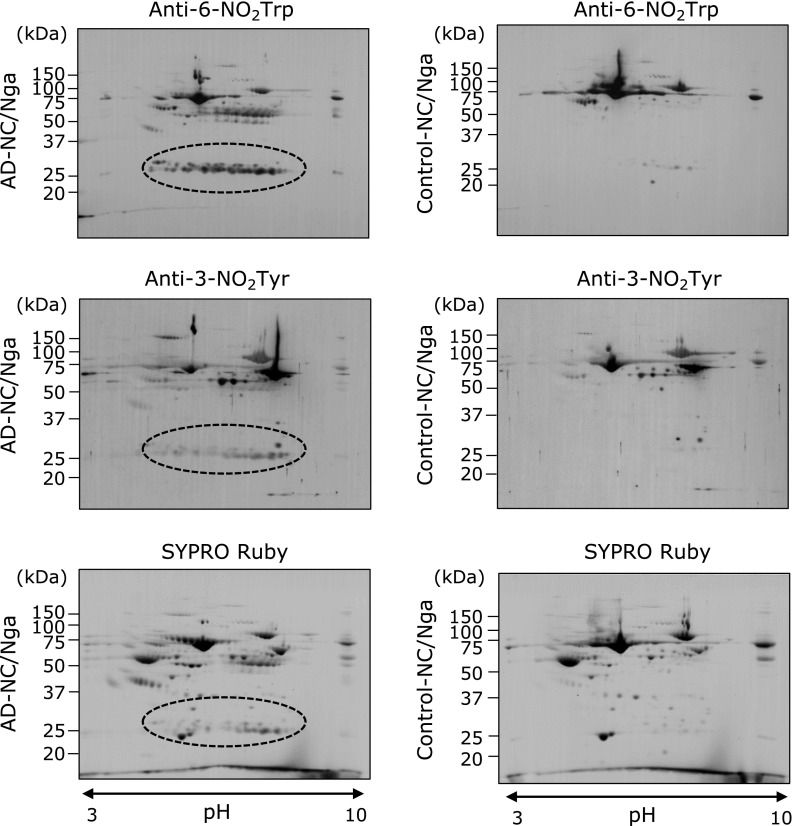
Nitrated plasma proteins detected by 2D-western blot analysis. The plasma of AD-NC/Nga mice (10-week-old NC/Nga mice bred under conventional conditions) and control-NC/Nga mice (10-week-old NC/Nga mice bred under SPF conditions) was separated by 2D-PAGE and subjected to western blot analysis. Left, plasma from AD-NC/Nga mice; right, plasma from control-NC/Nga mice. Top, immunoreactivity with anti-6-NO_2_Trp monoclonal antibody; middle, immunoreactivity with anti-3-NO_2_Tyr monoclonal antibody; and bottom, SYPRO Ruby-stained membranes. Spots surrounded by a dashed line showed a large difference between AD-NC/Nga and control-NC/Nga mice compared with the other spots.

We examined the generation of 6-NO_2_Trp and 3-NO_2_Tyr in the plasma proteins of AD-NC/Nga mice and control-NC/Nga mice by using 2D-western blot analysis. Signals for 6-NO_2_Trp were detected in the plasma proteins of both AD-NC/Nga and control-NC/Nga mice (Fig. 1, top). Among these signals, there was a large difference between the spots from the AD-NC/Nga mice compared with those from the control-NC/Nga mice (see spots surrounded by the dashed line in Fig. 1). A similar large difference between spots was also observed for the 3-NO_2_Tyr signals from the AD-NC/Nga mice and the control-NC/Nga mice (Fig. 1, middle). The bottom panel of Fig. 1 shows protein spots detected by SYPRO Ruby staining of the plasma proteins. The CBB-stained gel spots that corresponded to the spots surrounded by the dashed line in Fig. 1 were subjected to LC-MS/MS analysis and identified as immunoglobulin kappa-chains (data not shown).

### Semi-quantification of 6-NO_2_Trp and 3-NO_2_Tyr in the immunoglobulin light chain

**Fig. 2:**
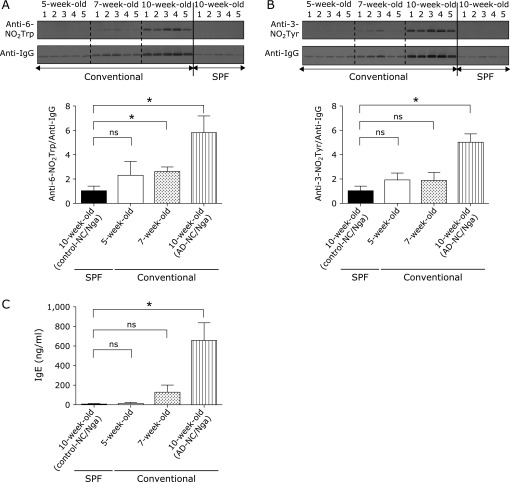
Semi-quantification of 6-NO_2_Trp and 3-NO_2_Tyr in the immunoglobulin light chain. Nitrated residue content was semi-quantitatively assessed by means of one-dimensional western blot analysis using anti-6-NO_2_Trp antibody (A), anti-3-NO_2_Tyr antibody (B), and anti-IgG antibody. Densitometry analysis is shown below. Data are presented as means ± SD of the relative quantity calculated from three experiments. Plasma IgE concentration was measured with an ELISA (C). Significance was analyzed by using a one-way ANOVA with Dunnett’s post-hoc test (*p<0.05, compared with control-NC/Nga group; ns, not significant); *n* = 5 animals per group.

We semi-quantitatively estimated the 6-NO_2_Trp and 3-NO_2_Tyr content in the immunoglobulin light chain in the plasma by using 1D-western blot analysis. We used plasma from 5-, 7-, and 10-week-old NC/Nga mice kept under conventional conditions and the 10-week-old control-NC/Nga mice. The signals for 6-NO_2_Trp and 3-NO_2_Tyr were detected on the membrane at sites consistent with the location of Ig light chains (Fig. 2A and B, upper panels). The signal intensities were normalized to the signal intensity of Ig light chains, detected by an anti-IgG antibody. We observed a statistically significant increase in the amount of 6-NO_2_Trp and 3-NO_2_Tyr present in the Ig light chain of 10-week-old AD-NC/Nga mice (Fig. 2A and B, lower panels). More importantly, we also observed a significant increase in the amount of 6-NO_2_Trp present in the Ig light chain of 7-week-old NC/Nga mice kept under conventional conditions, which was 2.6 times higher than that of the control-NC/Nga mice (Fig. 2A, lower panel). In contrast, the 3-NO_2_Tyr content of the Ig light chain was not statistically significantly different in the 7-week-old mice. We also observed a significant increase in the IgE levels in the plasma of 10-week-old NC/Nga mice maintained under conventional conditions (AD-NC/Nga mice) compared with age-matched control-NC/Nga mice, but did not find a similar significant difference between 7-week-old NC/Nga mice kept under conventional conditions and the control-NC/Nga mice (Fig. 2C).

### Identification of the nitrated amino acids in Ig light chains

**Fig. 3:**
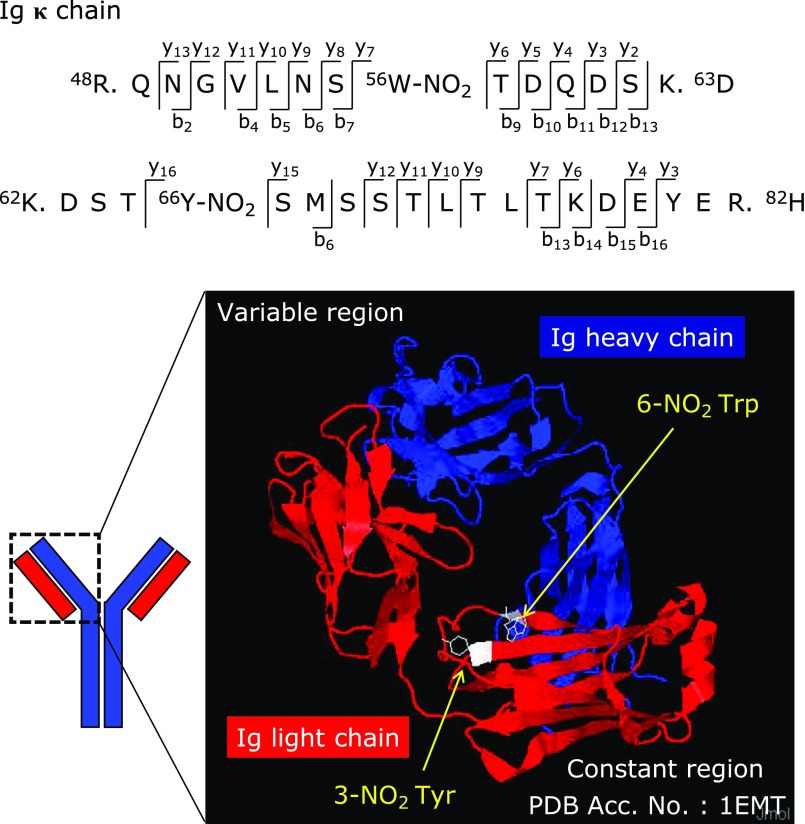
Identification of the nitrated amino acids in the Ig light chain. Nitration of Trp56 and Tyr66 in the constant region of the Ig kappa chain was identified by LC-MS/MS analysis. W-NO_2_ indicates an NO_2_Trp residue and Y-NO_2_ indicates an NO_2_Tyr residue. The lower figure shows a 3-dimensional model of immunoglobulin. The data for the 3D model were downloaded from the Protein Data Bank (PDB). The PDB accession number of the protein is 1EMT. The red color indicates the Ig light chain and the blue color indicates the Ig heavy chain. Trp56 and Tyr66 in the constant region of the Ig kappa chain, which were nitrated in this study, are shown in white.

We identified the positions of the nitrated tryptophan and nitrated tyrosine residues in the Ig light chains. We then carried out LC-MS/MS analysis of the trypsin-digested protein samples isolated from the bands that corresponded to the Ig light chains after separation by 1D-PAGE. We identified Trp56 and Tyr66 in the constant regions of the Ig kappa chain as the nitrated amino acids (Fig. 3, upper panel). The positions of the nitrated residues in the three-dimensional structure model of the Ig kappa chain are indicated in the lower panel of Fig. 3.

### Detection of NOS, SOD, 6-NO_2_Trp, and 3-NO_2_Tyr in the skin of NC/Nga mice

**Fig. 4:**
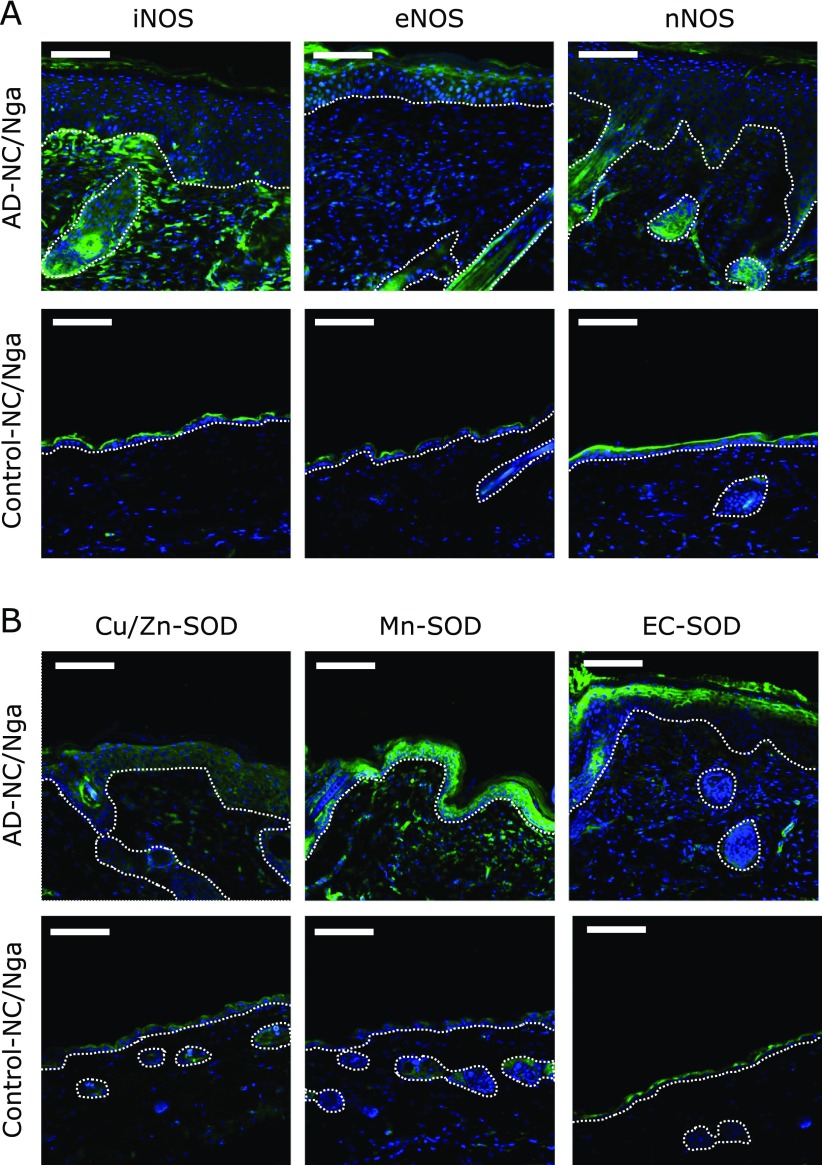
Immunofluorescence staining of the three isoforms of NOS (A) and the three isoforms of SOD (B). Skin sections were stained with the respective antibodies (green), and DNA was counterstained with DAPI (blue). The dashed line indicates the border between the dermis and the epidermis, whereas the closed or invaginated dashed line shows a cross-section of the hair and hair follicle. AD-NC/Nga, lesional skin from AD-NC/Nga mice; control-NC/Nga, normal skin from control-NC/Nga mice. Scale bars, 100 µm.

To evaluate the origin of the nitrated Ig light chain, we focused on the lesioned skin of the AD-NC/Nga mice. Since the nitration reaction is known to be caused by peroxynitrite, which is generated by the reaction of NO^•^ and superoxide, we first looked for the presence of nitric oxide synthase (NOS) and superoxide dismutase (SOD) in the skin samples. The localization of the three isoforms of NOS and the three isoforms of SOD was investigated by immunofluorescence staining of skin sections from AD-NC/Nga mice and control-NC/Nga mice. An intense immunofluorescence signal for iNOS was detected in the dermal skin samples from the 10-week old AD-NC/Nga mice relative to that in the normal samples from the control-NC/Nga mice (Fig. 4A). In contrast, there was no clear difference in the signals for eNOS or nNOS between the AD-NC/Nga and control-NC/Nga mouse dermal samples. The epidermis in the AD-NC/Nga mice comprised a thick layer of keratinocytes that greatly differed from that of the control-NC/Nga mice and prevented us from effectively comparing the signals in the epidermis. These histopathological changes in AD-NC/Nga mice are referred to as hyperkeratosis and acanthosis.^(16)^

As with iNOS, the immunofluorescence of Mn-SOD was stronger in the dermal skin samples from the AD-NC/Nga mice compared with that from the normal samples from the control-NC/Nga mice (Fig. 4B). In contrast, the signals for Cu/Zn-SOD and EC-SOD showed no clear differences. As for the epidermis, we were able to detect the Mn-SOD and EC-SOD signals in the samples from the AD-NC/Nga mice. These results indicate that NO^•^ production and oxidative stress were increased in the lesional dermis of the AD-NC/Nga mice.

**Fig. 5:**
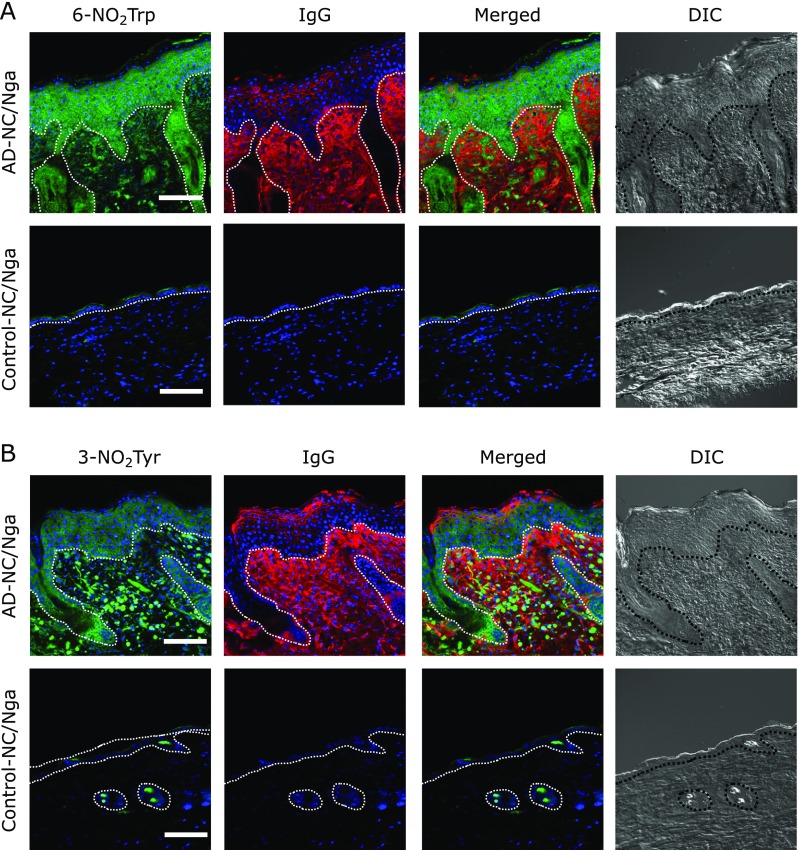
Immunofluorescence staining for 6-NO_2_Trp and 3-NO_2_Tyr. The skin sections were double-stained with anti-6-NO_2_Trp antibody (green) and anti-IgG antibody (red); DNA was counterstained with DAPI (blue) (A). 3-NO_2_Tyr was detected by using anti-3-NO_2_Tyr antibody (green) with anti-IgG antibody (red) and DAPI (blue) (B). Merged images indicate partial co-localization of 6-NO_2_Trp/3-NO_2_Tyr and IgG (yellow). DIC indicates differential interference contrast images. Scale bars, 100 µm.

We next examined the presence of 6-NO_2_Trp- and 3-NO_2_Tyr-containing proteins in the lesional skin of AD-NC/Nga mice by using immunofluorescence staining. Signals for 6-NO_2_Trp and 3-NO_2_Tyr were detected in the lesional dermis and epidermis of AD-NC/Nga mice (Fig. 5). The IgG signal was detected mainly in the lesional dermis of the AD-NC/Nga mice. This signal partially overlapped with the 6-NO_2_Trp and 3-NO_2_Tyr signals in the dermis. No signal was detected in the normal dermis of the control-NC/Nga mice. These results suggest that at least a portion of the 6-NO_2_Trp and 3-NO_2_Tyr in Ig light chains is generated in the lesional dermis of NC/Nga mice.

## Discussion

In this study, we sought new biomarkers for early stage AD by focusing on 6-NO_2_Trp and 3-NO_2_Tyr, which are known to be generated at sites of oxidative and nitrative stress.^(13,24)^ We used NC/Nga mice as an animal model for AD. Although some biomarkers in blood for oxidative stress has been proposed, such as nonmercaptalbumin,^(25)^ these biomarkers has not been studied for AD.

When 2D-western blot analyses were performed using plasma from AD-NC/Nga mice and control-NC/Nga mice, we found many positive spots indicative of 6-NO_2_Trp and 3-NO_2_Tyr, respectively, for both types of NC/Nga mice (Fig. 1). We identified the spots that were significantly increased in AD-NC/Nga mice as Ig light chains by using LC-MS/MS analyses (Fig. 1). Although a few other spots were increased in the AD-NC/Nga mice compared with the control mice (and remain to be analyzed), we focused on the Ig light chain as a candidate biomarker for AD. For this purpose, we measured the amounts of nitrated Ig light chain that were generated in 5-week-old, 7-week-old (before the onset of AD symptoms), and 10-week-old (after the onset of AD symptoms) NC/Nga mice. The amount of 6-NO_2_Trp and 3-NO_2_Tyr present in the Ig light chain increased with age (Fig. 2A and B). Importantly, we observed that the 6-NO_2_Trp content of the Ig light chain of 7-week-old NC/Nga mice was statistically significantly greater than that of the control mice. The IgE concentration, which is accepted as a biomarker for AD, in the plasma of the NC/Nga mice was also statistically significantly different from that of the control at 10-weeks old, but no statistically significant difference was observed at 7-weeks old (Fig. 2C). These results suggest that the generation of 6-NO_2_Trp in Ig light chain precedes the onset of AD symptoms. In contrast, the 3-NO_2_Tyr content of the Ig light chain did not show the same pattern of changes as that of 6-NO_2_Trp (Fig. 2B, bottom panel).

Arfat *et al.*^(26)^ recently reported the nitration and oxidation of IgG by *in vitro* addition of peroxynitrite, which is a reaction product of nitric oxide and superoxide. They showed that IgG was modified to form nitrotyrosine, nitrotryptophan, and dityrosine by the peroxynitrite addition, which caused structural perturbations. However, *in vivo* formation of nitrotryptophan and nitrotyrosine has not previously been reported. In fact, our study provides not only the first evidence of nitrotryptophan and nitrotyrosine in IgG *in vivo*, but also identifies the positions of the nitrated amino acids in the IgG. We identified the positions of the nitrated tryptophan and tyrosine residues in the amino acid sequence of the Ig light chain of AD-NC/Nga mice, by using proteomic analyses, as nitro-Trp56 and nitro-Tyr66 (Fig. 3). These nitrated amino acids are located near the variable region side of the constant region in the light chain. Therefore, the nitration of these residues could affect the antigen selectivity of the IgG. Further studies are required to clarify this possibility.

It is assumed that NO^•^ generation is elevated in patients with AD, because serum concentrations of NO_3_^−^, which is the reaction product of NO^•^ and molecular oxygen, are higher in these patients than in healthy control subjects.^(27)^ One study did detect 3-NO_2_Tyr in the skin of NC/Nga mice that were kept under conventional conditions.^(28)^ We found that 6-NO_2_Trp and 3-NO_2_Tyr were formed in the skin of AD-NC/Nga mice but not in the skin of control mice.^(14)^ Previously, we showed that 6-NO_2_Trp is formed in the skin of AD patients but not in the skin of healthy control subjects.^(14)^ Taken together, our findings suggested that NO^•^ and superoxide are generated simultaneously in the lesional skin of AD patients and AD model mice. To test this possibility, we examined the expression and localization of three different kinds of nitric oxide synthase. We found that the iNOS signal was increased in the lesional dermis of AD-NC/Nga mice compared with control mice. In contrast, there were no clear differences in the eNOS and nNOS signals in the dermis of these mice (Fig. 4A). A previous study using western blot analysis reported that eNOS expression in lesioned skin was increased; however, we did not find any clear differences in eNOS expression in the lesional dermis of our mice (Fig. 4A).^(28)^ To investigate the state of oxidative stress, we examined the expression of three SODs. The expression of Mn-SOD was increased significantly in the lesional dermis of the AD-NC/Nga mice (Fig. 4B, middle). However, we did not see a clear increase in the Cu/Zn-SOD signal or the EC-SOD signal in the dermis. Because it was difficult to compare the signals in the epidermis of the AD-NC/Nga mice, due to hyperkeratosis and acanthosis, the possibility remains that the Mn-SOD and EC-SOD signals were increasing. Since Mn-SOD is known to be induced under oxidative stress,^(29)^ our findings suggest that oxidative stress is increased in the lesional dermis of AD-NC/Nga mice.

Finally, to investigate the localization of 6-NO_2_Trp and 3-NO_2_Tyr, we tried to detect both signals. These signals were detected in the lesional dermis and epidermis, and partially overlapped with the IgG signal in the dermis (Fig. 5). Our results show that the generation of 6-NO_2_Trp and 3-NO_2_Tyr in the Ig light chain occurs at least in part in the dermis of lesional skin and may be carried to the plasma of AD-NC/Nga mice. Other possible sites for 6-NO_2_Trp and 3-NO_2_Tyr formations in the Ig light chain remain to be elucidated.

In conclusion, our study revealed that the 6-NO_2_Trp content of Ig light chain could be used as a new biomarker for detecting AD at an early stage. A portion of the 6-NO_2_Trp in Ig light chain may be generated in the lesional skin of AD-NC/Nga mice. In order to apply this potential biomarker to AD patients, studies on plasma from AD patients are now underway in our laboratory.

## Author Contribution

KI designed the study and wrote the initial draft of the manuscript. HK participated in the study design and performed data analysis. AS carried out the LC-MS/MS analysis and interpreted the data. MT and AO carried out the immunofluorescence staining. AK and YK contributed to provide materials. KT participated in the study design. FY participated in the study design and helped draft the manuscript.

## Abbreviations

AD: atopic dermatitis
BPB: bromophenol blue
CBB: Coomassie Brilliant Blue R-250
Cu/Zn-SOD: copper and zinc-containing superoxide dismutase
DAPI: 4',6-diamidino-2-phenylindole
DIC: differential interference contrast microscopy
DTT: dithiothreitol
EC-SOD: extracellular superoxide dismutase
ELISA: enzyme-linked immunosorbent assay
eNOS: endothelial nitric oxide synthase
IEF: isoelectric focusing
Ig: immunoglobulin
iNOS: inducible nitric oxide synthase
IPG: immobilized pH gradient
LC-ESI-MS/MS: liquid chromatography-electrospray ionization tandem mass spectrometry
Mn-SOD: manganese superoxide dismutase
nNOS: neuronal nitric oxide synthase
6-NO_2_Trp: 6-nitrotryptophan
3-NO_2_Tyr: 3-nitrotyrosine
PAGE: polyacrylamide gel electrophoresis
PBS: phosphate buffered saline
PBS-T: PBS containing 0.05% Tween 20
SPF: specific pathogen-free
